# Obstructive Sleep Apnea and Its Influence on Intracranial Aneurysm

**DOI:** 10.3390/jcm13010144

**Published:** 2023-12-27

**Authors:** Tae Young Jung, Eunkyu Lee, Minhae Park, Jin-Young Lee, Yun Soo Hong, Juhee Cho, Eliseo Guallar, Sang Duk Hong, Yong Gi Jung, Seonhye Gu, Jae Wook Ryoo, Eun-Yeon Joo, Je Young Yeon, Gwanghui Ryu, Hyo Yeol Kim

**Affiliations:** 1 Department of Otorhinolaryngology, Maryknoll Hospital, Busan 48972, Republic of Korea; 2 Department of Otorhinolaryngology-Head and Neck Surgery, Sungkyunkwan University School of Medicine, Samsung Medical Center, Seoul 06351, Republic of Koreabeans73@naver.com (M.P.); ent.jyg@gmail.com (Y.G.J.); 3 Health Promotion Center, Samsung Medical Center, Seoul 06351, Republic of Korea; godbeloved.lee@samsung.com; 4 Department of Epidemiology, Welch Center for Prevention, Epidemiology, and Clinical Research, Johns Hopkins Bloomberg School of Public Health, Baltimore, MD 21205, USA; 5 Department of Clinical Research Design and Evaluation, Samsung Advanced Institute for Health Science and Technology, Sungkyunkwan University, Seoul 06355, Republic of Korea; jh1448.cho@samsung.com; 6 Center for Clinical Epidemiology, Samsung Medical Center, Sungkyunkwan University School of Medicine, Seoul 06351, Republic of Korea; 7 Department of Digital Healthcare, Samsung Advanced Institute for Health Science and Technology, Sungkyunkwan University, Seoul 06355, Republic of Korea; 8 Biostatistics and Clinical Epidemiology Center, Samsung Medical Center, Sungkyunkwan University School of Medicine, Seoul 06351, Republic of Korea; higuseonhye@gmail.com; 9 Department of Radiology, Sungkyunkwan University School of Medicine, Samsung Medical Center, Seoul 06351, Republic of Korea; 10 Department of Neurology, Sleep Center, Samsung Medical Center, Samsung Biomedical Research Institute, Sungkyunkwan University School of Medicine, Seoul 06351, Republic of Korea; eunyeon1220.joo@samsung.com; 11 Department of Neurosurgery, Samsung Medical Center, Sungkyunkwan University School of Medicine, Seoul 06351, Republic of Korea; yeonjay.youn@samsung.com

**Keywords:** obstructive sleep apnea, intracranial aneurysm, polysomnography

## Abstract

Obstructive sleep apnea syndrome (OSAS) is associated with cerebrovascular disease, which can lead to life-threatening outcomes. The purpose of the study was to investigate the relationship between OSAS and comorbid intracranial aneurysms. We retrospectively reviewed 564 patients who underwent a polysomnography and brain magnetic resonance angiography as part of their health checkup. We calculated the prevalence of an intracranial aneurysm and OSAS in patients and measured the size of the intracranial aneurysm if present. The mean patient age was 55.6 ± 8.5 years, and 82.3% of them were men. The prevalence of an intracranial aneurysm in patients with OSAS was 12.1%, which is significantly higher than patients with non-OSAS (5.9%, *p* = 0.031). Patients with OSAS had a much higher prevalence of intracranial aneurysms, after adjusting all possible confounding factors such as age, sex, smoking status, alcohol drinking, and body mass index (odds ratio: 2.32; 95% confidence interval: 1.07–5.04). Additionally, the OSAS group had noticeably larger aneurysms compared with those of the non-OSAS group (3.2 ± 2.0 mm vs. 2.0 ± 0.4 mm, *p* = 0.013). We found a significant association between OSAS and intracranial aneurysms. OSAS could be another risk factor for the development of intracranial aneurysms.

## 1. Introduction

Obstructive sleep apnea syndrome (OSAS) is one of the most prevalent sleep-related breathing disorders, affecting 5% to 14% of the middle-aged population [1]. OSAS is well known to cause a wide variety of adverse health outcomes, especially cardiovascular and cerebrovascular diseases [2]. OSAS is also an important public health issue due to excessive daytime sleepiness that affects quality of life, work performance, and traffic accidents [3]. Cardiovascular or cerebrovascular diseases associated with OSAS, such as strokes and myocardial infarctions, potentially have life-threatening consequences that require immediate medical or surgical attention [4,5]. For this reason, the early detection and appropriate treatment of OSAS are important to reduce cardiovascular and cerebrovascular morbidity or mortality [6]. While the correlations between OSAS and cardiovascular diseases are well known, some links between OSAS and cerebrovascular diseases are less well understood.

Vascular aneurysms, such as aortic or intracranial aneurysms, are a major health problem and can be potentially fatal. Despite advances in treatment, a ruptured aneurysm is linked to significant mortality and morbidity rates [7]. Several studies have also approved a strong relationship between intracranial aneurysms and cardiovascular accidents. For patients with an aneurysm in the posterior circulation, the rupture risk increases up to 14.5% per year [7]. An estimated 5% to 15% of strokes are related to ruptures of intracranial aneurysms [8]. The mechanisms for the formation of an aneurysm have not been fully elucidated, but previous studies have suggested hemodynamic, genetic, and environmental factors; inflammation; and degeneration of the intracranial artery as possible mechanisms [9,10,11].

A screening test for OSAS was performed on patients with abdominal aortic aneurysms [12]. As a result, 40.5% of the patients had an apnea–hypopnea index (AHI) of 10 or higher. However, there have been no previous studies that evaluated the direct association between OSAS and intracranial aneurysms. Herein, we sought to investigate an association between OSAS and the prevalence and size of intracranial aneurysms.

## 2. Materials and Methods

### 2.1. Study Population

**Figure 1:**
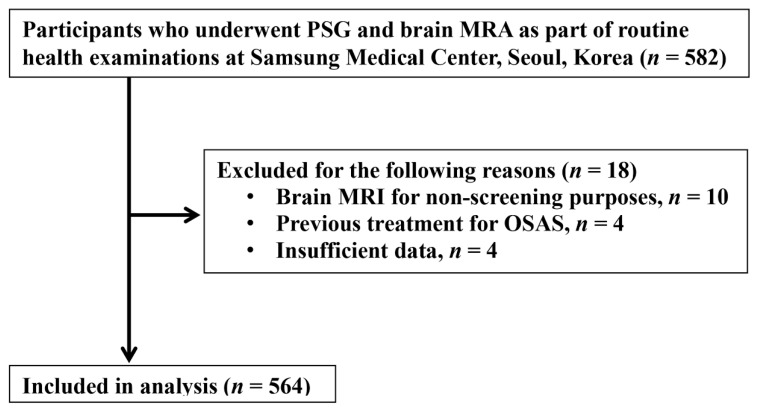
A flowchart of study participants. MRA: magnetic resonance angiography; MRI: magnetic resonance image; OSAS: obstructive sleep apnea syndrome; PSG: polysomnography.

From August 1997 to April 2012, we performed a cross-sectional study on 582 patients who had both a polysomnography (PSG) and brain magnetic resonance angiography (MRA) for the aim of completing a health checkup. For each participant, brain imaging for cerebral aneurysms using MRA was conducted within three months of PSG monitoring. Participants chose and underwent each test based on their own health status. We excluded 18 participants with the following characteristics: underwent brain MRI for medical, nonscreening purposes (*n* = 10), previously treated for OSAS (*n* = 4), and insufficient information in the medical record or PSG (*n* = 4). The final analysis included 564 participants (Figure 1). The institutional review board of the Samsung Medical Center approved this study (IRB No. 2015-02-036).

### 2.2. Health Examination

During the health screening program, all participants filled out a self-administered health questionnaire and underwent a comprehensive physical examination. The participants were questioned on their history of smoking and alcohol consumption; past medical history of diabetes, hypertension, hyperlipidemia, coronary heart disease, or cerebrovascular disease; and family history of cerebrovascular accidents (CVA).

At the time of screening, anthropometric measurements were performed by trained personnel. On the day of the screening exam, height, weight, and abdominal girth were measured. The calculation of the body mass index (BMI) was achieved by dividing the measured weight (kg) by the height squared (m^2^). Blood pressure was measured following a standardized protocol. An overnight fasting blood sample was collected at the antecubital vein to determine lipid and glucose levels.

Smoking history was divided into three categories: current, past, and never. Current alcohol intake was defined as more than one drink per week. History of ischemic heart disease and CVA was defined based on a self-reported physician diagnosis. At least one first-degree relative diagnosed with a stroke was considered to have a family history of CVA.

### 2.3. Polysomnography

Polysomnography monitoring was conducted using a standardized protocol. The presence of OSAS was confirmed with an overnight polysomnography (Alice3; Healthdyne Technologies^®^, Marietta, GA, USA). Four-channel electroencephalogram (EEG; C3/A2, C4/A1, O1/A2, and O2/A1), electro-oculogram, chin and anterior tibialis electromyograms, electrocardiogram, abdominal and thoracic movements by inductive plethysmography, airflow (nasal/oral), and oxygen saturation by pulse oximetry (SpO2) were recorded during the test.

Apnea and hypopnea were defined using a standard scoring system according to previous studies, and arousals were scored according to the rules of the American Sleep Disorders Association [13,14]. The AHI was calculated as the number of apnea and hypopnea arousals per hour divided by the total sleep time. The lowest SpO2 and arousal index were obtained. The severity of OSAS was categorized by the AHI as non-OSA (AHI < 5/h), mild (5 ≤ AHI < 15/h), moderate (15 ≤ AHI < 30/h), and severe (AHI ≥ 30/h).

### 2.4. Intracranial Aneurysm Image Analysis

**Figure 2:**
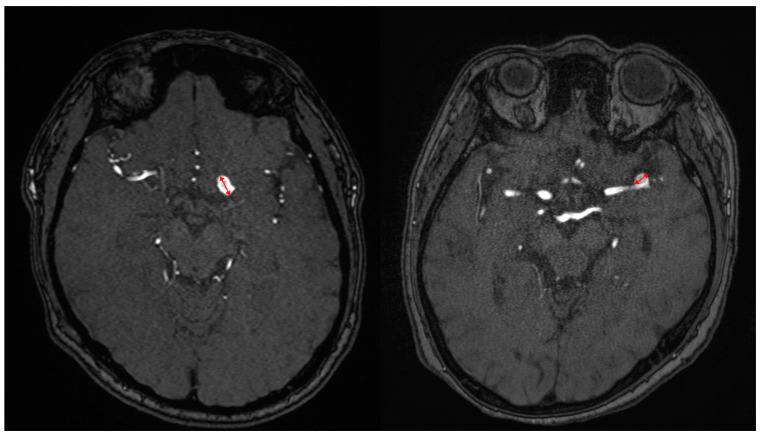
Maximal diameter measurements of the intracerebral aneurysm (red arrows).

For all cases, the angiographic characteristics of aneurysms were directly measured from digital subtraction of the MRA, which included the size and location of the aneurysm as described by a radiologist. The following parameters were used to perform the 3D MRA: echo time = 6.9 msec; repetition time = 27 msec; flip angle = 20°; slab thickness = 48 mm; effective section thickness = 0.8 mm; matrix = 512 × 256; and field of view = 20 cm. An aneurysm was considered present if there was a clear separation between the aneurysm neck and the parent artery (Figure 2). The maximum length on the transverse plane of the axial source picture was used to determine the size of the intracranial aneurysm.

### 2.5. Statistical Analysis

Statistical analysis was carried out using the one-way analysis of variance (ANOVA) or Wilcoxon rank–sum test for comparison of continuous variables, and the χ^2^ test or Fisher’s exact test was used for comparison of discrete variables, as appropriate. In this study, the continuous variables are expressed as the mean and standard deviation. The odds ratios and 95% confidence intervals (CI) for the association between OSAS and prevalent cerebral aneurysms were estimated using three models of logistic regression analyses. The crude odds ratio was estimated in the initial model (model 1). Model 2 was adjusted for age and sex. Model 3 was further adjusted for potential confounders, including current smoking status, current alcohol intake status, and BMI [15].

Statistical analyses were executed using STATA version 14 (StataCorp LP, College Station, TX, USA). A value of *p* < 0.05 was considered statistically significant.

## 3. Results

A total of 564 participants were included in the final analysis. The mean age was 55.6 (±8.5) years and the majority (82.3%) were men. Study participants were categorized by the degree of OSAS based on the AHI obtained from PSG monitoring (Table 1). Among them, 412 (73.0%) patients were diagnosed with OSAS (AHI ≥ 5). Those with a higher degree of OSAS were more likely to be men, current smokers, and current drinkers compared to those without OSAS. Also, a higher degree of OSAS was associated with a higher BMI, lower high-density lipoprotein (HDL) level, higher triglyceride level, higher prevalence of hypertension, and stronger family history of CVA.

A total of 59 patients with an asymptomatic intracranial aneurysm and 505 subjects without an intracranial aneurysm were enrolled in the present study. Among the 59 patients with intracranial aneurysms, 46 had saccular-shaped aneurysms (78.0%), and the remaining 13 had fusiform- or broad neck-shaped aneurysms. Between the two groups, there was no statistical difference in the shape or location of aneurysms (Table 2). The most frequent site for an intracranial cerebral aneurysm was the internal carotid artery (69.5%), followed by the anterior communicating artery (11.9%). The location of the intracranial aneurysm did not reveal a statistically significant difference when comparing the presence of OSAS. The overall prevalence of intracranial aneurysms was higher in participants with OSAS (12.1%) compared with those without OSAS (5.9%, *p* = 0.031).

In the univariate analysis (model 1), the odds ratio for the association between OSAS and intracranial aneurysms was 2.19 (95% CI, 1.05–4.58) (Table 3). To account for any potential confounders in the association, a multivariable logistic regression analysis was performed. After adjusting for age and sex, the association remained significant with an odds ratio of 2.37 (95% CI, 1.11–5.07). In a model that further adjusted for current smoking or alcohol intake status and BMI (model 3), the adjusted odds ratio for intracranial aneurysms in those with OSAS compared to those without did not change significantly (OR, 2.32; 95% CI, 1.07–5.04).

There was also a significant difference in the intracranial aneurysm size between the non-OSAS group and the OSAS group. The OSAS group had significantly larger aneurysms (3.2 ± 2.0 mm) than those of the non-OSAS group (2.0 ± 0.4 mm; *p* = 0.013).

## 4. Discussion

Although there have been several studies that demonstrated the relationship between OSAS and intracranial aneurysms, this is the first study to investigate the positive association between OSAS and intracranial aneurysms in a large number of routine health examination participants [16,17,18,19]. As subjects in this study were patients who had undergone a brain MRA for health screening purposes, it is noteworthy that this study reports the prevalence of asymptomatic intracranial aneurysms in OSAS patients.

OSAS and unruptured intracranial aneurysms share common risk factors, including smoking, alcohol intake, and hypertension. In our data, the OSAS group exhibited a higher proportion of current smokers, individuals with high alcohol intake, and those with hypertension compared to the non-OSAS group (Table 1). These shared risk factors may act as confounding variables influencing the prevalence of intracranial aneurysms. However, a distinction in the gender distribution exists between these two diseases. While being a man is a risk factor for OSAS, being a woman is a risk factor for intracranial aneurysms [20,21]. A multivariate analysis was conducted to account for these effects.

In this study, the prevalence of intracranial aneurysms in OSAS patients was 12.1%, which is significantly higher than in non-OSAS patients (5.9%). Regarding the prevalence of unruptured aneurysms in the Korean general population is 2.23% [22], OSAS patients showed a definitely higher prevalence of intracranial aneurysms in this study. Though there have been no studies that reported the exact prevalence of intracranial aneurysms in OSAS patients, Zaremba et al. reported that OSAS patients have a higher incidence of subarachnoid hemorrhage (0.15%) than patients with other sleep disorders (0.02%, *p* = 0.04) [23]. A subarachnoid hemorrhage is mainly caused by ruptured intracranial aneurysms, suggesting that OSAS increases the risk of intracranial aneurysms. The baseline characteristics of patients with an intracranial aneurysm were comparable to those in a larger series of patients with unruptured intracranial aneurysms in terms of age distribution, cardiovascular risk factor profiles, size, shape, and location of the intracranial aneurysm [10,24,25].

Neagos et al. investigated the structural properties and metabolic characteristics of OSAS in the Romanian population, finding that triglyceride levels were correlated with the AHI in the mild OSAS group, while no correlation was observed between triglyceride levels and the AHI in the severe OSAS group [26,27]. In our results, the triglyceride levels tended to be higher in the severe OSAS group, but there was no statistical significance which coincides with other reports.

OSAS elicits cycles of hypoxia, CO_2_ retention, and increased activity of the sympathetic nervous system. Hypoxia and hypercapnia act synergistically to heighten any sympathetic activity [28]. This phenomenon induces oscillation in the cardiac vagal and sympathetic activity, and as a result, it changes the heart rate and blood pressure [29]. In addition, OSAS is associated with systemic inflammation. Several studies have reported increased plasma concentrations of inflammatory markers including cytokines (interleukin-6 and tumor necrosis factor-α), acute-phase proteins, matrix metalloproteinases, the erythrocyte sedimentation rate, and C-reactive protein in OSAS patients [30,31]. Moreover, patients with OSAS have high plasma concentrations of endothelial adhesion molecules and reduced nitric oxide availability, suggesting that inflammation and vascular endothelial dysfunction can induce the development of a vascular disease [32]. Inflammation in response to OSAS is assumed to contribute to the formation of intracranial aneurysms.

Furthermore, our findings suggest that patients with OSAS have larger-sized intracranial aneurysms than patients without OSAS. In combination with the higher prevalence of intracranial aneurysms in patients with OSAS, this finding provides clues that OSAS may be causally associated with the development of intracranial aneurysms, which needs further research for the evaluation of causation.

Although the prevalence of intracranial aneurysms was clearly increased in mild and moderate OSAS patients after adjusting for potential confounders, the association was not seen in the severe OSAS group. As patients with moderate to severe OSAS are more likely to have a larger intracranial aneurysm, they were also more likely to experience symptoms and may have been excluded from our study population, which only included asymptomatic participants who underwent a brain MRA as a screening exam. Also, the limited number of patients with severe OSAS was insufficient to obtain a reliable estimation of the effect.

There are some limitations to this study. Our study showed a higher prevalence of intracranial aneurysms observed by MRA (10.5%) compared to previous studies. In previous studies, the prevalence of unruptured intracranial aneurysms was estimated to be between 0.4 and 6% [33]. The predominance of middle-aged men among the patients enrolled in this study may have contributed to these findings. Second, since this study was conducted in a cross-sectional manner, we were unable to establish the causal relationship between OSAS and intracranial aneurysms, which would require the analysis of incident events in a prospective cohort study. Third, there were more male participants enrolled in this study, and the results may not be generalizable to women. Due to the fact that men are considered a risk factor in obstructive sleep apnea, it is presumed that there were more men among those who chose PSG in their health examination, as they may be relatively more inclined to select this examination. Finally, as our study included patients from a single tertiary care center, our results may not be applicable to the general population.

There are a few strengths of this study. It is the first to find an association between OSAS and intracranial aneurysms, and it includes a large number of participants who underwent both a PSG and brain MRA, which allows objective diagnoses of OSAS and intracranial aneurysms, respectively. As our study population is composed of asymptomatic patients who underwent a brain MRA for screening purposes, it represents the subclinical population. Most previous studies include patients who were admitted to a hospital with an aneurysm and did not include people in the subclinical stage of intracranial aneurysms.

## 5. Conclusions

In conclusion, we identified a relationship between OSAS and intracranial aneurysms. A higher prevalence of intracranial aneurysms was observed in patients with OSAS compared to those without OSAS. Furthermore, patients with OSAS displayed larger aneurysm sizes. Our findings have a potential clinical significance in that patients who have OSAS may need to be screened for the presence of an intracranial aneurysm. We propose that OSAS may be another important risk factor influencing the development and enlargement of intracranial aneurysms. Further studies into the effects of OSAS on intracranial aneurysms are needed to evaluate its clinical significance.

## Figures and Tables

**Table 1:** Baseline participant characteristics and prevalence of intracranial aneurysm by severity of obstructive sleep apnea.

Characteristics	Non-OSA (*n* = 152)	Obstructive Sleep Apnea (OSA)		
		Mild (*n* = 155)	Moderate (*n* = 120)	Severe (*n* = 137)
Age	54.6 ± 8.8	56.4 ± 8.4	55.3 ± 8.5	55.9 ± 8.2
Sex ^a^				
Male ^a^	103 (67.8)	127 (81.9)	104 (86.7)	130 (94.9)
Female	49 (32.2)	28 (18.1)	16 (13.3)	7 (5.1)
BMI (kg/m^2^) ^a^	23.8 ± 2.6	24.6 ± 2.6	25.4 ± 2.5	26.3 ± 2.9
Smoking				
Never smoker (%) ^a^	74 (48.7)	100 (64.5)	85 (70.8)	96 (70.1)
Current smoker (%) ^a^	26 (17.1)	39 (25.2)	34 (28.3)	39 (28.5)
Current alcohol intake (%) ^a^	96 (63.2)	119 (76.8)	93 (77.5)	119 (86.9)
HDL-C, mg/dL ^a^	54.7 ± 15.3	52.0 ± 12.3	48.8 ± 11.4	49.1 ± 11.9
Comorbidity				
HTN(%) ^a^	43 (28.3)	49 (31.6)	44 (36.7)	61 (44.5)
DM (%)	13 (8.6)	23 (14.8)	17 (14.2)	19 (13.9)
Dyslipidemia (%)	43 (28.3)	42 (27.1)	40 (33.3)	43 (31.4)
IHD (%)	7 (4.6)	44 (7.1)	7 (5.8)	10 (7.3)
History of CVA (%)	7 (4.6)	8 (5.2)	7 (5.8)	8 (5.8)
Family history of CVA (%)	40 (26.3)	43 (27.7)	28 (23.3)	43 (31.4)
Intracranial aneurysm				
Yes	9 (5.9)	22 (14.2)	18 (15.0)	10 (7.3)
No	143(94.1)	133 (85.8)	102 (85.0)	127 (92.7)

Values are mean ± SD, median (interquartile range), or number (%). ^a^ *p* < 0.05, Statistically significant difference between non-OSA and OSA. AHI: apnea–hypopnea index; BMI: body mass index; CVA: cerebrovascular accident; OSA: obstructive sleep apnea; SD: standard deviation.

**Table 2:** Differences in the characteristics of intracranial aneurysms between the obstructive sleep apnea and non-obstructive sleep apnea group.

Variables	Total (*n* = 564)	Non-OSA (*n* = 152)	OSA (*n* = 412)	*p*-Value
Intracranial aneurysm	59 (10.5)	9 (5.9)	50 (12.1)	0.031 *
Shape				
Saccular	46 (78.0)	7 (77.8)	39 (78)	1.000
Fusiform or broad neck	13 (22.0)	2 (22.2)	11 (22)	
Location				
Internal carotid artery	41 (69.5)	6 (66.7)	35 (70)	0.069
Anterior cerebral artery	3 (5.1)	1 (11.1)	2 (4.0)	1.000
Anterior communicating artery	7 (11.9)	1 (11.1)	6 (12.0)	0.681
Middle cerebral artery	5 (8.5)	1 (11.1)	4 (8.0)	1.000
Posterior communicating artery	3 (5.1)	0	3 (6.0)	0.567

Values are mean numbers (%). * *p* < 0.05: statistically significant difference between non-OSA and OSA. OSA: obstructive sleep apnea.

**Table 3:** Association between obstructive sleep apnea and aneurysms.

	Prevalent Aneurysm *N* (%)	Model 1 OR (95% CI)	Model 2 OR (95% CI)	Model 3 OR (95% CI)
OSA (−)	9 (5.9)	1.00 (reference)	1.00 (reference)	1.00 (reference)
OSA (+)	50 (12.1)	2.19 (1.05–4.58)	2.37 (1.11–5.07)	2.32 (1.07–5.04)

Model 1: unadjusted; model 2: adjusted for age and sex; model 3: further adjusted for current smoking, alcohol intake, and body mass index. CI: confidence interval; OR: odds ratio; OSA: obstructive sleep apnea. (−): patient without disease, (+): patient with disease.

## Data Availability

The data presented in this study are available on request from the corresponding author.

## Abbreviations

OSAS	Obstructive sleep apnea syndrome
PSG	Polysomnography
CVA	Cerebrovascular accident
EEG	Electroencephalogram
AHI	Apnea–hypopnea index
